# Effects of task-based teaching on middle school students’ motivation in physical education: a quasi-experimental study based on self-determination theory

**DOI:** 10.3389/fpsyg.2026.1886581

**Published:** 2026-08-05

**Authors:** Di Zhu, Xianglong Jiang, Chengxin Li, Tongtong Che

**Affiliations:** School of Physical Education, Qingdao University, Qingdao, Shandong, China

**Keywords:** basic psychological needs, middle school students, physical education motivation, self-determinant theory, tasked-based learning and teaching

## Abstract

This study adopted self-determination theory as the theoretical framework and used a quasi-experimental design to examine the effects of task-based teaching on middle school students’ basic psychological needs satisfaction and motivation in basketball learning. A total of 102 eighth-grade students from a junior high school in Qingdao were randomly assigned to a task-based teaching group, a traditional teaching group, and a blank control group, with a 6-week teaching intervention. Results showed that task-based teaching significantly improved students’ autonomy, competence, and relatedness (all large effects). It also significantly enhanced intrinsic motivation and identified regulation while reducing amotivation, leading to a more adaptive motivational profile. Correlational analyses indicated that autonomy was the core predictor of motivational internalization and reduced amotivation. These findings confirm that task-based teaching effectively enhances the quality of students’ motivation in physical education by satisfying basic psychological needs, with no significant gender differences.

## 1 Introduction

Insufficient physical activity has become a major challenge in global public health. According to the Global Status Report on Physical Activity released by the World Health Organization (2022), approximately 42 million deaths each year are directly related to a lack of regular physical activity, a figure that accounts for a substantial proportion of global mortality. This problem is particularly serious among children and adolescents. Based on a pooled analysis of 298 population-based surveys in 65 countries around the world, Guthold et al. (2020) found that more than 80% of adolescents aged 11–17 years fail to meet the recommendation of 60 min of moderate to vigorous physical activity per day. Physical inactivity over the long term can not only cause physiological (reduced cardiorespiratory fitness and higher prevalence of obesity) but also have adverse effects on the mental health, cognitive development and social adaptation, thereby hindering the development of lifelong exercise habits.

As the main setting for adolescents’ physical activity, the middle school physical education classroom plays a central role in teaching motor skills, developing physical literacy, and promoting health-related behaviors. A large body of evidence suggests that adolescents’ participation in physical activity is closely associated with their motivational level. A meta-analysis by Owen et al. (2014) showed that adolescents with higher autonomous motivation engaged in extracurricular physical activity more frequently and for longer durations than those with lower motivation. A local study by Xiang (2013) also found that motivation quality was the strongest psychological variable predicting persistence in physical exercise among middle school students. However, participation in current middle school physical education classes is often uneven, and passive learning remains common. Against the background of increasing academic pressure, mandatory course arrangements alone are no longer sufficient to stimulate students’ sustained willingness to participate. Therefore, how to optimize classroom teaching methods to stimulate and maintain students’ motivation for physical education from a psychological perspective has become a key issue in the reform of middle school physical education.

Self-Determination Theory (SDT) offers one of the most systematic frameworks for understanding how motivation develops in physical education. Beyond the classical dichotomy of motivation (versus no motivation) SDT suggests that motivation is a continual phenomenon in the spectrum of external regulation to internalized identification. Autonomous motivation, symbolized mainly by the intrinsic motivation and identified regulation is considered an important predictor of long-lasting participation and profound engagement. This theoretical supposition has been generally confirmed in the field of school physical education and in adolescent exercise environments (Chen et al., 2014; Ntoumanis and Standage, 2009; Standage et al., 2006). Deci and Ryan (2000) defined the main place of the three fundamental psychological needs, which are autonomy, competence, and relatedness. Among the earlier to apply this framework to Western middle school physical education classrooms were Ntoumanis (2001) and Standage et al. (2003) who demonstrated the direct effect that classroom settings had on need satisfaction and motivational development. A meta-analysis of 112 studies by Vasconcellos et al. (2020) further supported the idea of the cross-cultural applicability of the theory in the Chinese school physical education context.

Chinese researchers have also developed a series of localized research on the association between autonomous motivation and physical education learning outcomes. Cao (2010) was one of the first ones who studied the internalization mechanism of physical education learning motivation through the lens of SDT. The effect of the basic psychological need satisfaction on exercise behavior among middle school students was verified by Ding and Mao (2014). Hu et al. (2020), in a systematic review, clearly pointed out that the degree of internalization of autonomous motivation is a core psychological variable determining the quality of physical education teaching. Mechanistically, SDT underlines that motivation is not produced particularly in response to extrinsic rewards or punishments, but it is naturally produced when the basic psychological needs of individuals are being fulfilled. As the classroom setting helps students to make an autonomous choice, develop competencies, and feel socially connected, their motivation to learn is likely to change towards a higher quality. This perspective holds that the structural aspect of the classroom, such as task arrangement and patterns of interaction, have a direct influence on the psychological experience and motivation levels of the students.

In recent years, increasing attention has been paid to the effects of classroom interventions on the trans-contextual transfer of motivation. Zhu and Mao (2025) found that teachers’ autonomy-supportive behaviors in physical education not only enhanced students’ autonomous motivation within the classroom, but also promoted their leisure-time physical activity through trans-contextual effects. Zhu and Yin (2017) further confirmed that the need for support from significant others, such as parents and teachers, is a key factor in promoting the internalization of adolescents’ exercise motivation. Wang and Liu (2024), in their analysis of student incentive measures in European comprehensive school physical activity programs, also suggested that SDT-based intervention strategies can effectively transform physical education participation from school-based requirements into extracurricular habits. These studies provide a solid theoretical and empirical foundation for the present study’s focus on classroom task design as a core element in exploring effective ways to enhance middle school students’ motivation in physical education.

Against this background, task-based teaching, which uses tasks as the core organizational unit, demonstrates unique application value. This teaching method is oriented toward clear learning objectives. By creating authentic movement contexts, designing progressively structured learning tasks, and guiding students to engage in cooperative inquiry, it enables students to achieve understanding and skill acquisition through “learning by doing.” Unlike the one-way model of traditional teaching, in which teachers demonstrate, students imitate, and skills are repeatedly practiced, task-based teaching places greater emphasis on students’ agency and process experience. Existing studies have preliminarily confirmed its positive effects in physical education. Yao (2008) found that scientifically designed classroom learning tasks significantly enhanced situational interest in physical education among junior high school students. Wallhead and Ntoumanis (2004), in a curricular intervention study, showed that task-centered teaching could improve students’ motivational responses. Gaspar et al. (2021) further demonstrated that game-understanding-based task teaching improved elementary school students’ autonomous motivation.

In collective sports such as basketball, if technical practice, tactical understanding, teamwork, and evaluative feedback are integrated into clearly structured and goal-oriented learning tasks, students are more likely to experience autonomy, competence, and peer support during task completion, thereby promoting the internalization and stable development of learning motivation. This idea is highly consistent with the core features of a high-quality physical education classroom proposed by Ji et al. (2024), namely clear objectives, appropriate tasks, and active student participation. However, although task-based teaching has been fully verified in fields such as language education, its applicability and mechanisms in middle school physical education contexts remain insufficiently examined. Existing studies have focused mainly on the influence of teachers’ autonomy-supportive behaviors on motivation, while paying relatively limited attention to tasks as the central carrier of classroom teaching. In particular, how tasks influence learning motivation by acting on students’ basic psychological needs remains to be further explored. Therefore, there are three interrelated gaps in the existing literature. First, although task-based teaching has been widely discussed in the field of general education and has shown promise for application in physical education, its application in middle school basketball classes has yet to be fully validated through intervention designs. Second, physical education research based on Self-Determination Theory (SDT) often emphasizes teachers’ autonomy-supportive behaviors, while the tasks themselves—the specific vehicles through which students practice skills, collaborate, and receive feedback—have received relatively little empirical attention. Third, the potential link between the task-based teaching approach, the fulfillment of basic psychological needs, and motivation to learn basketball has not yet been clearly explored within a coherent classroom model. Therefore, the conceptual framework of this study is as follows. Task-based instruction is expected to provide students with greater autonomy, more challenging practice opportunities, and opportunities for collaborative interaction. These classroom experiences correspond to the three basic psychological needs proposed by Self-Determination Theory (SDT): autonomy, competence, and relatedness. In turn, higher levels of need fulfillment are expected to be associated with more adaptive forms of basketball learning motivation, including higher levels of intrinsic motivation and identity regulation, as well as lower levels of amotivation. Since this study employs correlational analysis rather than mediation analysis or structural equation modeling, this pathway is regarded as a theoretical guide for interpretation rather than a formally tested causal mechanism.

Based on this framework, this study addresses four research questions: (1) Over a six-week period, does task-based instruction alter students’ autonomy, sense of competence, and sense of belonging in basketball learning? (2) Does task-based instruction alter students’ intrinsic motivation, identity regulation, external regulation, and amotivation? (3) Are there gender differences in the effects of the intervention? (4) What are the relationships between the three basic psychological needs and different types of basketball learning motivation?

## 2 Participants and methods

### 2.1 Participants

In this study, students in the junior high school of Qingdao in China at the eighth grade were selected to investigate the effects of task-based teaching on the motivation of middle school students in learning basketball. Based on self-determination theory, the study further analyzed students’ psychological changes and motivational development during basketball learning. To help make the study scientifically rigorous and scientifically feasible, 108 students were recruited and stratified by sex and age, and then randomly assigned to one of three teaching groups: a task-based teaching group (experimental group), a traditional teaching group (comparison group), and a non-basketball physical education group (blank control group), with 36 students in each group.

All participants volunteered to take part in the study and provided informed assent after receiving information about the study purpose, procedures, and possible risks; guardian consent was also obtained. Participants were allocated at the individual level rather than by intact original classes. Specifically, after eligibility screening, students were stratified by sex and then randomly assigned by a computer-generated random-number table to one of three teaching conditions: task-based teaching, traditional teaching, or a blank-control condition, with 36 students initially allocated to each group. During the study, six participants withdrew for personal reasons, leaving 102 participants who completed the full intervention and post-test assessment.

This study was approved by the Ethics Committee of the Medical College of Qingdao University (Ethics No. QDU-HEC-2025574). All participants and their guardians were fully informed of the study purpose, procedures, and possible risks, and participation was voluntary based on informed consent. Privacy protection and ethical standards were strictly followed throughout the study. All personal information and test data were coded and archived to ensure confidentiality and data security.

Inclusion criteria were as follows: (1) being an eighth-grade student; (2) being in good health without serious chronic diseases, such as cardiovascular or neurological disorders; (3) having no history of long-term medication use and no participation in other physical fitness or mental health interventions; (4) being able to participate in all teaching sessions on time; and (5) undergoing basketball skill screening before the experiment, including basic skills such as passing, dribbling, and shooting. Based on the screening results, students were assigned to the experimental group, comparison group, and blank control group. Before the intervention, the three groups were generally comparable in basketball skill level, physical condition, and sport participation, with no statistically significant differences between groups. The main difference between groups was the form of teaching intervention: the experimental group received task-based teaching, the comparison group received traditional teaching, and the blank control group did not receive specialized basketball instruction.

Exclusion criteria were as follows: (1) having serious chronic disease or motor impairment; (2) having received systematic physical training or intervention within the previous month; and (3) being unable to complete the full intervention, such as withdrawal or frequent absence.

### 2.2 Experimental design and intervention program

#### 2.2.1 Experimental design

This study adopted a pretest–posttest quasi-experimental design with three groups: an experimental group receiving task-based teaching, a comparison group receiving traditional teaching, and a blank control group receiving no specific basketball teaching intervention but participating in other regular physical education activities. This design was selected because the intervention was implemented in a real school physical education setting, where complete control over students’ daily learning environment, peer interaction, and school timetable was not feasible. Compared with a single-group pretest–posttest design, the inclusion of both a traditional-teaching comparison group and a blank-control group made it possible to distinguish changes associated with task-based basketball teaching from changes related to regular physical education exposure, repeated measurement, or maturation. Compared with a fully controlled laboratory experiment, the present design had higher ecological validity for evaluating an instructional approach within routine school practice. The intervention lasted 6 weeks and included three stages: pretest, teaching intervention, and posttest. The pretest was performed 1 week prior to the intervention (Week 0), and the posttest was performed immediately after the intervention (Week 6) to assess the effects of teaching activities. The two sessions of 45 min each were scheduled in the week of the 6-week intervention period, with the experimental and comparison groups receiving two 45-min sessions each week, and the blank control group being exposed to other regular physical education activities without basketball-specific instructions.

#### 2.2.2 Intervention program

The six-week intervention was organized according to a progressive basketball-learning sequence. To improve transparency and reproducibility, the intervention procedures and the contrast between teaching conditions are summarized in Table 1. In this study, task-based teaching was operationally defined as a student-centered instructional approach in which each lesson was organized around a goal-directed basketball task that required students to solve a movement or tactical problem through exploration, peer cooperation, decision-making, practice, and feedback. The defining pedagogical features were: (1) using a meaningful task as the core unit of learning; (2) embedding technical practice in game-like or problem-solving situations; (3) providing students with limited but real choices in strategies, roles, or movement solutions; (4) encouraging cooperation and peer communication; and (5) using teacher feedback mainly as scaffolding rather than as direct command. By contrast, traditional teaching was characterized by teacher explanation and demonstration, uniform technical drills, repeated imitation, and teacher-led correction. Therefore, the key distinction between the two conditions was not only the weekly basketball content, but also the instructional logic through which the content was delivered.

**Table 1:** Summary of the six-week intervention procedures and differences between teaching conditions.

Week	Week objective	Task-based teaching group	Traditional teaching group	Blank-control group
1	Ball handling:passing/catching	Goal task: maintain continuous passes in pairs/small groups; students adjust spacing, timing, and passing choice with peer feedback.	Teacher demonstration and explanation followed by unified passing/catching drills and correction.	Regular PE without basketball-specific instruction.
2	Dribbling:shooting	Small-group dribbling route and shooting challenge; students select routes and compare completion strategies.	Straight-line dribbling, cone dribbling, and set shooting through teacher-led drills.	Regular PE without basketball-specific instruction.
3	Basic cooperation	Two-on-two cooperation tasks, including simplified pick-and-roll situations; emphasis on communication and movement choice.	Fixed cooperation routes practiced after teacher explanation and demonstration.	Regular PE without basketball-specific instruction.
4	Decision-making in attack	Triple-threat tasks requiring students to decide whether to pass, shoot, or drive according to defensive cues.	Similar technical elements practiced through teacher-led explanation, demonstration, and repetition.	Regular PE without basketball-specific instruction.
5	Small-sided game application	Three-on-three half-court games with task constraints, such as requiring at least three passes before shooting.	Conventional half-court games and teacher-directed technical review.	Regular PE without basketball-specific instruction.
6	Integrated application	Students design and demonstrate offensive combinations and explain task strategies.	Comprehensive technical review and teacher-directed teaching games.	Regular PE without basketball-specific instruction.

### 2.3 Measure

#### 2.3.1 Psychological measure

This study selected basic psychological need satisfaction and basketball learning motivation as the key psychological outcome variables. Because both instruments were contextually adapted for basketball learning, the manuscript reports the original scale sources, the adaptation logic, sample items, and internal consistency estimates in the present sample. The adaptations were limited to contextual wording and were not treated as newly validated scales.

Basketball learning motivation was measured using the Sport Motivation Scale-II (SMS-II; Pelletier et al., 2013), which was developed from the original Sport Motivation Scale (SMS; Pelletier et al., 1995). The SMS-II has demonstrated acceptable construct validity and reliability in previous sport-context validation studies. In the present study, the scale was minimally adapted to the basketball learning context by replacing general sport-related wording with basketball-specific phrasing, such as “basketball learning” or “basketball class,” while retaining the original theoretical meaning and response format. Four dimensions relevant to the present research questions were used: intrinsic motivation, identified regulation, external regulation, and amotivation, with 18 items in total. Responses were recorded on a 7-point Likert scale, with higher scores indicating stronger endorsement of the corresponding motivation type. Sample adapted items included: “I learn basketball because it is enjoyable” (intrinsic motivation), “I learn basketball because I think it is important for me” (identified regulation), “I learn basketball because others expect me to do so” (external regulation), and “I do not really know why I learn basketball” (amotivation). The adaptation was limited to contextual wording and did not change the dimensional structure of the scale. In the current sample, the overall Cronbach’s *α* was 0.801, and the *α* coefficients of the four dimensions were 0.925 for intrinsic motivation, 0.899 for identified regulation, 0.823 for external regulation, and 0.930 for amotivation. These reliability results, together with evidence from previous validation studies, support the use of the adapted scale in the present exploratory classroom intervention. However, no confirmatory factor analysis was conducted, and the psychometric performance of the basketball-adapted version should be further examined in future studies with larger samples.

Basic psychological need satisfaction was assessed using the Basic Psychological Needs in Exercise Scale (BPNES; Vlachopoulos and Michailidou, 2006), a 12-item measure developed to assess autonomy, competence, and relatedness satisfaction in exercise contexts. The scale consists of three dimensions, with four items for each dimension. In the present study, the items were contextually adapted for basketball learning in physical education by replacing general exercise wording with basketball-class wording while preserving the original conceptual content. Responses were recorded on a 7-point Likert scale, with higher scores indicating greater satisfaction of the corresponding psychological need. Sample adapted items included: “In basketball class, I feel that I can make choices about how to complete learning tasks” (autonomy), “In basketball class, I feel capable of completing the learning tasks” (competence), and “In basketball class, I feel connected with my classmates” (relatedness). In the present sample, the overall Cronbach’s *α* was 0.914. The α coefficients for the three dimensions were 0.824 for autonomy, 0.852 for competence, and 0.887 for relatedness, indicating good internal consistency. Because the scale was contextually adapted, the results should be interpreted as evidence of internal reliability rather than a full validation of the modified version.

### 2.4 Statistical analysis

Data organization and statistical analysis were carried out with the help of Excel 2016 and SPSS 26.0. All the data are provided in terms of mean ± standard deviation (*M* ± SD).

The pretest scores, posttest scores and the pre and post change scores of each variable were tested in terms of normality before formal statistical analysis using the KolmogorovSmirnov test and the ShapiroWilk test. The findings revealed that some dimensions such as autonomy, competence, relatedness, intrinsic motivation, identified regulation, external regulation, and amotivation had statistical significance (*p* < 0.01) indicating some deviations of normal distribution. Considering that normality tests are sensitive to minor deviations when the sample size is relatively large, and that all variables in this study were continuous scale scores, repeated-measures analysis of variance is robust to a certain degree of non-normality when group sizes are relatively balanced and no severe outliers are present. Therefore, after reporting the results of the normality tests, repeated-measures ANOVA was used to examine the effects of the teaching intervention. To reduce the possible influence of non-normality, a logarithmic transformation was performed for clearly skewed variables as a sensitivity analysis. In case the significance judgments were the same before and after the transformation, the means and standard deviations of the original scores are reported in the main text to allow the interpretation of the practical significance of teaching.

Baseline comparability across the three groups was examined before the intervention for basketball skill level, physical condition, and sport participation. Normality was assessed using the Shapiro–Wilk test, and homogeneity of variance was assessed using Levene’s test. When the assumptions of normality and homogeneity of variance were satisfied, one-way ANOVA was used to compare the three groups; otherwise, the Kruskal–Wallis H test was applied. These analyses were used to describe comparability on measured baseline characteristics and were not interpreted as evidence of complete equivalence across all possible pre-intervention characteristics.

The specific statistical model was a 3 (group: experimental, comparison, blank control) × 2 (sex: male, female) × 2 (time: pretest, posttest) repeated-measures ANOVA. Basic psychological needs (autonomy, competence, and relatedness) and basketball learning motivation (intrinsic motivation, identified regulation, external regulation, and amotivation) were the dependent variables. Because the time factor included only two levels, the sphericity assumption was automatically satisfied; therefore, Mauchly’s test of sphericity was not conducted. The ANOVA focused on the main effects of group and time, as well as the group × time interaction. Sex was included as a between-subjects variable to examine whether the intervention effects differed by sex. When interactions reached statistical significance, simple effects analyses were further conducted to compare pretest–posttest differences within each group and group differences at each time point. All multiple comparisons were corrected using the Bonferroni method to control the risk of Type I error.

To evaluate the practical effects of the teaching intervention, partial η^2^ was reported as the effect size indicator for ANOVA. Effect size was interpreted according to Cohen’s criteria: partial η^2^ = 0.01 as a small effect, 0.06 as a medium effect, and 0.14 as a large effect. For within-group pretest–posttest changes and pairwise between-group comparisons, Cohen’s d was further calculated to quantify the practical significance of intervention effects, using the following criteria: *d* = 0.2 as a small effect, 0.5 as a medium effect, and 0.8 as a large effect. By reporting both partial η^2^ and Cohen’s d, this study comprehensively evaluated the effect of task-based teaching from both statistical and practical perspectives.

In addition, Spearman’s rank correlation coefficients were used to examine associations between basic psychological need satisfaction and basketball learning motivation. Specifically, rank correlations were calculated between autonomy, competence, and relatedness and the four motivation dimensions: intrinsic motivation, identified regulation, external regulation, and amotivation. These analyses were used to describe theoretically informed relationships among the study variables. Because correlation analysis cannot establish causal mechanisms or mediating pathways, the results were not interpreted as evidence that psychological need satisfaction mediated the effect of task-based teaching on motivation. The significance level for all statistical tests was set at *p* < 0.05.

## 3 Results

### 3.1 Baseline comparability of measured characteristics

Before the intervention, baseline comparisons were conducted for basketball skill level, physical condition, and sport participation. As shown in Table 2, no statistically significant differences were observed among the task-based teaching group, traditional teaching group, and blank-control group in basketball skill level, physical condition, or sport participation (all *p* > 0.05). These results indicate that the three groups were broadly comparable on the measured baseline characteristics before the intervention.

**Table 2:** Baseline comparability of measured characteristics before the intervention.

Baseline variable	Task-based teaching group (*n* = 34)	Traditional teaching group (*n* = 34)	Blank-control group (*n* = 34)	Test	Statistic	*p* value	Interpretation
Basketball skill level	13.57 ± 1.48	13.07 ± 1.56	13.64 ± 1.09	ANOVA	*F* = 1.6439	0.1984	n.s.
Physical condition	50.12 ± 4.52	49.61 ± 4.97	50.27 ± 4.66	ANOVA	*F* = 0.1806	0.8350	n.s.
Sport participation	50.97 ± 7.46	49.21 ± 5.74	49.82 ± 6.84	Kruskal–Wallis	H = 1.0556	0.5899	n.s.

Values are presented as mean ± standard deviation. ANOVA, one-way analysis of variance; n.s., not significant. The Kruskal–Wallis H test was used for sport participation because the assumptions for parametric testing were not fully satisfied.

Repeated-measures ANOVA showed that all three psychological need indicators had highly significant main effects of time: autonomy, *F* = 57.36, *p* < 0.001; competence, *F* = 43.46, *p* < 0.001; and relatedness, *F* = 25.40, *p* < 0.001. This indicates that, overall, students’ satisfaction of psychological needs improved significantly after teaching. The main effect of group was significant for autonomy (*F* = 6.46, *p* = 0.002), competence (*F* = 3.70, *p* = 0.028), and relatedness (*F* = 4.99, *p* = 0.009), indicating overall differences between groups. The group × time interaction was highly significant for all three indicators: autonomy, *F* = 20.61, *p* < 0.001; competence, *F* = 13.37, *p* < 0.001; and relatedness, *F* = 7.92, *p* = 0.001. This suggests that intervention effects differed significantly across groups. The main effect of sex and all sex-related interactions were not significant (*p* > 0.05), indicating that the effects of task-based teaching were consistent for male and female students. This suggests that the observed changes in psychological need satisfaction did not differ significantly by sex.

Further within-group analyses showed that autonomy increased significantly in both the experimental group (*p* < 0.001, *d* = 1.66, large effect) and the comparison group (*p* < 0.001, *d* = 0.50, medium effect), whereas no significant change was observed in the blank control group. In terms of competence, the experimental group (*p* < 0.001, *d* = 1.40, large effect) and the comparison group (*p* < 0.001, *d* = 0.43, small-to-medium effect) both showed significant improvement, while the blank control group did not. Relatedness also increased significantly in both the experimental group (*p* < 0.001, *d* = 1.19, large effect) and the comparison group (*p* < 0.001, *d* = 0.46, small-to-medium effect), while the blank control group showed no obvious change. The effect sizes in the experimental group reached large levels for all three psychological need dimensions (*d* = 1.19–1.66), indicating high practical significance. In contrast, traditional teaching in the comparison group produced only small-to-medium effects (*d* = 0.43–0.50), and no obvious changes occurred in the blank control group. These findings suggest that task-based teaching was associated with substantial within-group improvements in autonomy, competence, and relatedness. However, the between-group comparisons should be interpreted cautiously because the task-based group did not differ significantly from the traditional teaching group on these three dimensions.

Bonferroni-corrected multiple comparisons showed significant differences between the experimental group and the blank control group in autonomy (*p* = 0.002, *d* = 1.66, large effect), competence (*p* = 0.024, *d* = 1.40, large effect), and relatedness (*p* = 0.007, *d* = 1.19, large effect). Differences between the experimental and comparison groups were not significant for autonomy (*p* = 0.204), competence (*p* = 0.367), or relatedness (*p* = 0.697). Differences between the comparison and blank control groups were also not statistically significant for autonomy (*p* = 0.252), competence (*p* = 0.758), or relatedness (*p* = 0.169) (see Tables 3–5).

**Table 3:** Changes in basic psychological need satisfaction before and after the intervention and repeated-measures ANOVA results.

Variable	Group	Gender	Pre-intervention (M ± SD)	Post-intervention (M ± SD)	Main effects (partial-η^2^)			*F*		
					[Group]	[Time]	[Gender]	[Group] × [Time](partial η^2^)	[Gender] × [Time](partial η^2^)	[Group] × [Time] × [Gender](partial η^2^)
Autonomy	Experimental	Male	3.53 ± 1.07	4.49 ± 0.41	6.46** (0.12)	57.36*** (0.37)	0.02 (0.00)	20.61*** (0.30)	0.01 (0.00)	0.36 (0.01)
		Female	3.43 ± 0.78	4.47 ± 0.43						
	Control	Male	3.35 ± 1.00	3.69 ± 0.65						
		Female	3.65 ± 0.85	3.82 ± 0.66						
	Blank	Male	3.35 ± 1.10	3.45 ± 0.94						
		Female	3.12 ± 1.06	3.25 ± 0.81						
Competence	Experimental	Male	3.71 ± 1.09	4.43 ± 0.39	3.70* (0.07)	43.46*** (0.31)	0.32 (0.00)	13.37*** (0.22)	0.55 (0.01)	1.03 (0.02)
		Female	3.37 ± 0.87	4.45 ± 0.35						
	Control	Male	3.45 ± 1.10	3.80 ± 0.61						
		Female	3.63 ± 0.88	3.88 ± 0.74						
	Blank	Male	3.55 ± 1.04	3.63 ± 0.87						
		Female	3.29 ± 1.07	3.41 ± 0.83						
Relatedness	Experimental	Male	3.94 ± 0.96	4.49 ± 0.44	4.99** (0.09)	25.40*** (0.21)	0.04 (0.00)	7.92*** (0.14)	0.29 (0.00)	0.22 (0.01)
		Female	3.94 ± 0.78	4.57 ± 0.35						
	Control	Male	3.88 ± 0.95	4.02 ± 0.63						
		Female	4.00 ± 0.78	4.10 ± 0.75						
	Blank	Male	3.71 ± 1.01	3.77 ± 0.86						
		Female	3.41 ± 1.30	3.61 ± 0.98						

*p < 0.05; **p < 0.01; ***p < 0.001. M, mean; SD, standard deviation; partial η^2^, partial effect size. G, group; T, time; S, sex.

**Table 4:** Changes in basketball learning motivation before and after the intervention and repeated-measures ANOVA results.

Variable	Group	Gender	Pre-test (*M* ± SD)	Post-test (*M* ± SD)	Main-effect (Partial η^2^)			*F*		
					[Group]	[Time]	[Gender]	[Group] × [Time] (Partial η^2^)	[Gender] × [Time] (Partialη^2^)	[Group] × [Time] × [Gender] (Partialη^2^)
Intrinsic Motivation	Experimental	Male	5.01 ± 1.88	6.13 ± 0.69	2.71 (0.05)	18.56*** (0.16)	0.00 (0.00)	26.70*** (0.36)	0.14 (0.00)	0.75 (0.02)
		Female	4.93 ± 1.71	6.26 ± 0.53						
	Control	Male	4.82 ± 1.75	4.63 ± 1.31						
		Female	5.01 ± 1.49	4.69 ± 1.04						
	Blank	Male	4.90 ± 2.03	5.18 ± 1.69						
		Female	4.93 ± 1.72	4.93 ± 1.72						
Identified Regulation	Experimental	Male	5.22 ± 1.20	5.78 ± 0.91	0.71 (0.01)	6.24* (0.06)	0.65 (0.01)	22.56*** (0.32)	0.18 (0.00)	1.84 (0.04)
		Female	4.46 ± 1.64	5.43 ± 0.96						
	Control	Male	5.50 ± 1.31	5.15 ± 0.93						
		Female	5.00 ± 1.68	4.63 ± 1.31						
	Blank	Male	4.51 ± 1.89	4.74 ± 1.61						
		Female	5.06 ± 1.26	5.06 ± 1.26						
External regulation	Experimental	Male	5.04 ± 1.54	6.03 ± 0.85	3.87* (0.07)	16.92*** (0.15)	0.51 (0.01)	22.79*** (0.32)	0.18 (0.00)	0.65 (0.01)
		Female	4.29 ± 1.78	5.63 ± 0.74						
	Control	Male	4.66 ± 1.66	4.50 ± 1.23						
		Female	4.44 ± 1.31	4.21 ± 0.97						
	Blank	Male	4.38 ± 1.49	4.51 ± 1.35						
		Female	4.71 ± 1.50	4.65 ± 1.24						
Amotivation	Experimental	Male	3.18 ± 1.74	2.82 ± 0.38	0.89 (0.02)	4.21* (0.04)	0.67 (0.01)	23.83*** (0.33)	2.10 (0.02)	2.27 (0.05)
		Female	4.26 ± 1.75	2.75 ± 0.45						
	Control	Male	3.26 ± 2.03	4.49 ± 0.91						
		Female	2.88 ± 1.71	4.12 ± 0.64						
	Blank	Male	3.57 ± 2.23	4.07 ± 1.82						
		Female	2.78 ± 1.59	3.29 ± 1.23						

*p < 0.05; **p < 0.01; ***p < 0.001. M, mean; SD, standard deviation; partial η^2^, partial effect size. G, group; T, time; S, sex.

**Table 5:** Results of Spearman rank correlation analysis.

Variable	Intrinsic	Identified	External	Amotivation
Autonomy	0.295**	0.293**	0.297**	−0.259**
Compet	0.217*	0.158	0.167	−0.160
Related	0.157	0.123	0.219*	−0.095

*p < 0.05; **p < 0.01.

Repeated-measures ANOVA showed that task-based teaching had differentiated effects on the dimensions of students’ motivation in physical education. For intrinsic motivation, the main effect of time (*F* = 18.56, *p* < 0.001) and the group × time interaction (*F* = 26.70, *p* < 0.001) were significant, while the main effect of group was not significant (*F* = 2.71, *p* = 0.072). For identified regulation, the main effect of time (*F* = 6.24, *p* = 0.014) and the group × time interaction (*F* = 22.56, *p* < 0.001) were significant, while the main effect of group was not significant (*F* = 0.71, *p* = 0.494). For external regulation, the main effect of group (*F* = 3.87, *p* = 0.024), the main effect of time (*F* = 16.92, *p* < 0.001), and the group × time interaction (*F* = 22.79, *p* < 0.001) were all significant. For amotivation, the main effect of time (*F* = 4.21, *p* = 0.043) and the group × time interaction (*F* = 23.83, *p* < 0.001) were significant, while the main effect of group was not significant (*F* = 0.89, *p* = 0.415). The main effect of sex and all sex-related interactions were not significant across all dimensions (*p* > 0.05), suggesting that the intervention effects of task-based teaching were consistent across male and female students. This suggests that the observed motivational changes did not differ significantly by sex.

Further within-group analyses showed that intrinsic motivation increased significantly in the experimental group (*p* < 0.001, *d* = 0.75, medium effect), while no significant changes were observed in the comparison group (*d* = 0.13) or blank control group (*d* = 0.15). Identified regulation increased significantly in the experimental group (*p* < 0.001, d = 0.56, medium effect), while no significant changes occurred in the comparison group (*d* = 0.28) or blank control group (*d* = 0.07). External regulation increased significantly in the experimental group (*p* < 0.001, *d* = 0.80, large effect), decreased slightly in the comparison group (*d* = 0.17), and showed no significant change in the blank control group (*d* = 0.07). Amotivation decreased significantly in the experimental group (*p* < 0.001, *d* = 0.76, medium effect), whereas it tended to increase in the comparison group (*d* = 0.64, medium effect) and blank control group (*d* = 0.28, small effect). Overall, the experimental group showed medium-effect improvements in autonomous motivation, including intrinsic motivation and identified regulation, a large-effect increase in external regulation, and a medium-effect decrease in amotivation, forming a positive motivational pattern characterized by high autonomous motivation, moderate external regulation, and low amotivation.

Bonferroni-corrected multiple comparisons showed that the differences between the experimental and comparison groups were not significant for intrinsic motivation (*p* = 0.085) or identified regulation (*p* = 1.000), but were significant for external regulation (*p* = 0.032, d = 1.06, large effect). Differences between the experimental group and the blank control group were not significant for intrinsic motivation (*p* = 0.282), identified regulation (*p* = 0.719), or external regulation (*p* = 0.105). No significant differences were found between the comparison group and the blank control group across all dimensions (*p* > 0.05).

### 3.2 Correlation analysis

#### 3.2.1 Spearman rank correlation analysis

To examine the associations between students’ basic psychological needs (autonomy, competence, and relatedness) and basketball learning motivation (intrinsic motivation, identified regulation, external regulation, and amotivation), correlation analysis was conducted. Because the data did not conform to normal distribution, Spearman’s rank correlation was used to test the rank associations between variables.

The correlation results showed that autonomy was significantly and positively correlated with intrinsic motivation (*r* = 0.295, *p* < 0.01), identified regulation (*r* = 0.293, *p* < 0.01), and external regulation (*r* = 0.297, *p* < 0.01), and was significantly and negatively correlated with amotivation (*r* = −0.259, *p* < 0.01). Competence was significantly and positively correlated only with intrinsic motivation (*r* = 0.217, *p* < 0.05), whereas its correlations with identified regulation (*r* = 0.158, *p* > 0.05), external regulation (*r* = 0.167, *p* > 0.05), and amotivation (*r* = −0.160, *p* > 0.05) were not significant. Relatedness was significantly and positively correlated with external regulation (*r* = 0.219, *p* < 0.05), whereas its correlations with intrinsic motivation (*r* = 0.157, *p* > 0.05), identified regulation (*r* = 0.123, *p* > 0.05), and amotivation (*r* = −0.095, *p* > 0.05) were not significant.

These results suggest that autonomy was the psychological need most consistently associated with more self-determined motivational indicators and lower amotivation. Competence was significantly associated only with intrinsic motivation, suggesting that students who felt more capable in basketball learning tended to report greater interest and enjoyment. Relatedness was significantly associated with external regulation, suggesting that peer connection and social recognition may also be linked to more externally oriented reasons for participation in this classroom context. Because these were correlation results, they should be interpreted as associations rather than evidence of causal mechanisms or mediation pathways.

## 4 Discussion

### 4.1 Effects of task-based teaching on basic psychological need satisfaction

The results of this study show that task-based teaching significantly improved middle school students’ satisfaction of autonomy, competence, and relatedness needs in basketball learning, and that the intervention effects on all three dimensions were clearly superior to those of traditional teaching. This finding is highly consistent with the core proposition of self-determination theory and is also supported by the findings of Haerens et al. (2013) and Kalajas-Tilga et al. (2020). SDT posits that autonomy, competence, and relatedness are three innate basic psychological needs. When a teaching environment effectively supports these needs, individuals tend to show higher levels of learning engagement, positive emotions, and persistence. In the present study, the experimental group showed large-effect improvements in all three psychological need dimensions (*d* = 1.19–1.66), whereas the comparison group showed only small-to-medium effects (*d* = 0.43–0.50), and the blank control group showed no significant changes. This fully demonstrates the unique advantage of task-based teaching in satisfying students’ basic psychological needs, with high practical significance.

Regarding autonomy, the experimental group showed the largest improvement (*d* = 1.66), which is closely related to the design logic of task-based teaching that emphasizes students’ agency. Unlike the prescribed model of traditional teaching, where the teachers give a uniform demonstration, and the students conduct a uniform practice, task-based teaching allows the students the opportunity of a bit of choice by defining distinct but not narrowly defined tasks for students. For example, during passing practice, the students had the right to choose the methods and the routes of passing freely; during dribbling practice, the students had the right to design the challenge routes in groups; during tactical practice, the students had the right to decide their attacking modes freely. With the chance of making choices, students felt they were “the real owners of the learning,” not only an obedient “tool” under the guidance of the teachers, which largely satisfied the needs for autonomy, which is supported by Standage et al. (2005) on his findings that affording adequate choices and rewarding autonomy attempts would increase the needs satisfaction for autonomy in physical education class.

Regarding competence, students in the task-based teaching group showed a large within-group improvement (*d* = 1.40). Task-based teaching follows the design principle of progression from easy to difficult, decomposing complex basketball skills into a series of challenging but attainable subtasks. In completing each subtask, students can obtain immediate successful experiences and clearly observe their progress, gradually building confidence in their abilities. This interpretation is supported by previous SDT-based physical education studies. For example, Standage et al. (2005) found that need-supportive physical education environments predicted students’ basic psychological need satisfaction and intrinsic motivation, while Standage and Gillison (2007) reported that perceived competence and autonomy positively predicted autonomous motivation in school physical education. Wallhead and Ntoumanis (2004) also showed that a sport education curriculum could enhance students’ motivational responses partly through a more task-involving climate and stronger perceptions of autonomy and competence. However, evidence in the broader literature is not entirely consistent. Vasconcellos et al. (2020) reported substantial heterogeneity across SDT-based physical education studies, suggesting that the effects of need-supportive interventions may vary according to intervention fidelity, duration, age group, and measurement approach. Similarly, Fernández-Espínola et al. (2020) found that cooperative-learning interventions had a small-to-moderate positive effect on intrinsic motivation, but the evidence showed large heterogeneity and not all studies demonstrated significant between-group advantages. In the present study, the task-based group did not differ significantly from the traditional teaching group in competence after correction for multiple comparisons. Thus, the competence finding should be interpreted as a context-specific within-group improvement during the basketball unit rather than conclusive evidence that task-based teaching is universally superior to traditional instruction in enhancing perceived competence.

Regarding relatedness, the experimental group also showed a large-effect improvement (*d* = 1.19). Basketball is a collective sport, and task-based teaching further strengthens small-group cooperation in classroom organization. In this intervention, most tasks required students to work together in groups, such as group dribbling challenges, two-on-two pick-and-roll cooperation, and three-on-three half-court games. During these tasks, students had to communicate, negotiate, divide responsibilities, and support one another in solving problems. Such frequent positive interactions not only improve communication and teamwork skills, but also help students develop good peer relationships and enhance their sense of belonging and acceptance in class. Huhtiniemi et al. (2019) also noted that positive peer interaction in physical education is an important way to satisfy students’ relatedness needs, and satisfaction of relatedness further promotes classroom participation.

In addition, this study found that the main effect of sex and all interactions involving sex, group, and time were not significant. This suggests that task-based teaching promotes basic psychological need satisfaction similarly among male and female students. This suggests an important practical implication: The task-driven and collaborative inquiry teaching model is gender-neutral and applicable to both male and female students. This is in agreement with Sun et al. (2001), suggesting that the sex differences in adolescents’ physical activity motivation is not conclusive but has a close relationship to the classroom contexts. As long as the teaching method satisfies students’ basic psychological needs, it can produce similarly positive effects for both male and female students.

Overall, the findings are consistent with the theoretical logic linking task-based teaching to basic psychological need satisfaction, but they should be interpreted as exploratory evidence rather than proof of a causal psychological pathway. By reconstructing classroom task organization, task-based teaching may provide students with more opportunities for autonomous choice, competence challenge, and peer cooperation, which are conditions theoretically aligned with autonomy, competence, and relatedness satisfaction.

### 4.2 Effects of task-based teaching on basketball learning motivation

The results of this study suggest that task-based teaching was associated with favorable within-group changes in several dimensions of basketball learning motivation. Specifically, the task-based group showed significant within-group increases in intrinsic motivation and identified regulation, a significant reduction in amotivation, and an increase in external regulation. However, Bonferroni-corrected comparisons did not show significant differences between the task-based group and the traditional-teaching group for intrinsic motivation or identified regulation. Therefore, the motivational findings should be interpreted as promising evidence of change within the task-based condition rather than definitive evidence that task-based teaching was superior to traditional teaching across all motivational dimensions.

The significant enhancement in intrinsic motivation was a very noticeable outcome of task-based teaching (*d* = 0.75). Intrinsic motivation means engaging in an activity because one is interested in it, enjoys it, or finds it satisfying, and it’s a learning motivation at its highest quality. Instead of being a tedious and mechanical repetitive drill activity in the traditional teaching, task-based teaching situates the learning of basketball skills in real and interesting basketball activities. Students are no longer merely “practicing movements”; rather, they experience the enjoyment of learning through completing tasks, solving problems, and engaging in competition. For example, in dribbling practice, students completed dribbling tasks of different difficulty levels through a challenge format; in tactical practice, students applied learned tactics through two-on-two and three-on-three games. Such contextualized and game-like learning can effectively stimulate students’ curiosity and desire to explore, enabling them to gain pleasure and a sense of achievement from the activity itself, thereby significantly enhancing intrinsic motivation. This finding is consistent with Wallhead and Ntoumanis (2004) and Gaspar et al. (2021), who demonstrated that contextualized and problem-solving-based physical education activities effectively improve students’ intrinsic motivation.

The increase in identified regulation (*d* = 0.56) indicates that task-based teaching not only enhances the extent to which students “like learning,” but also strengthens their cognitive recognition that learning is “worthwhile.” Identified regulation refers to motivation based on recognizing the value and significance of an activity, and it is an important component of autonomous motivation. For junior high school students, learning motivation is not entirely dependent on interest; it is also closely related to their understanding of the value of the activity. Task-based teaching enables students to clearly recognize the importance of basketball learning for their own development by setting clear learning goals, demonstrating the practical value of skills, and emphasizing personal growth during the learning process. For example, during teaching, the teacher explained that basketball can improve physical fitness, cultivate teamwork, and relieve academic pressure. After task completion, students were guided to reflect on their progress and gains. As students gradually recognized the personal meaning of basketball learning, external learning requirements were progressively internalized into self-endorsed behavioral goals, reflected in increased identified regulation. This is consistent with SDT’s explanation of the internalization of extrinsic motivation (Deci and Ryan, 2000; Ryan and Deci, 2020).

Notably, the experimental group also showed a significant increase in external regulation (*d* = 0.80). From the ideal perspective of SDT, teaching interventions usually aim to enhance autonomous motivation rather than external regulation. However, considering the teaching context and intervention duration of this study, this result is reasonable. First, task-based teaching emphasizes goal achievement, peer evaluation, classroom demonstration, and competition performance. These elements may lead students, especially in the early stage of intervention, to pay greater attention to task completion, their performance within the group, and others’ evaluations, thereby increasing external regulation to some extent. Second, in school physical education, autonomous motivation and external regulation are not absolutely opposed, but represent different stages on the same motivational continuum. Moderate external regulation can serve as the starting point for motivational internalization. As teaching continues and students gain enjoyment and value recognition from the activity, external regulation may gradually transform into identified regulation and intrinsic motivation. Therefore, the increase in external regulation in this study should be viewed as a stage-specific manifestation of motivational internalization rather than as a negative outcome. Future studies may extend the intervention period to further observe dynamic changes in external regulation.

The reduction in amotivation (*d* = 0.76) is another finding with important practical value. Amotivation refers to a state in which individuals lack interest in and value recognition of an activity and do not believe they can complete it, leading to passive, perfunctory, or avoidant behaviors. Previous studies have shown that amotivation is one of the main reasons for insufficient participation in middle school physical education classes (Cheon et al., 2016; Vasconcellos et al., 2020). In the present study, amotivation decreased significantly in the experimental group, whereas it tended to increase in the comparison and blank control groups. In the present study, amotivation decreased significantly in the task-based group, whereas it tended to increase in the comparison and blank-control groups. This result may be related to the simultaneous provision of choice, attainable challenges, and peer support in the task-based condition, but causal explanation requires further mediation or longitudinal analysis.

Taken together, the motivational results suggest that task-based teaching may be useful for improving students’ engagement quality and reducing low-motivation tendencies in basketball learning. The correlation analysis below further clarifies how different dimensions of psychological need satisfaction were associated with different forms of learning motivation.

## 5 Conclusion and future directions

### 5.1 Conclusion

Guided by self-determination theory, this study examined whether a six-week task-based basketball teaching intervention was associated with changes in students’ basic psychological need satisfaction and basketball learning motivation, and whether these changes differed by gender.

The findings suggest that the six-week task-based basketball teaching intervention was associated with positive changes in students’ basic psychological need satisfaction and basketball learning motivation. Students in the task-based teaching group showed improvements in autonomy, competence, and relatedness, as well as more adaptive motivational changes, particularly higher intrinsic motivation and identified regulation and lower amotivation. Gender-related analyses further indicated that the intervention effects were not entirely uniform across male and female students, suggesting that gender differences should be considered when designing and evaluating task-based basketball instruction. In addition, the correlation results showed that autonomy, competence, and relatedness were generally associated with more self-determined forms of basketball learning motivation. However, because the present study used correlation analyses rather than mediation analysis or structural equation modeling, these relationships should be interpreted as theoretically meaningful associations rather than evidence of a confirmed causal mechanism. Taken together, task-based basketball teaching may be a promising approach for supporting students’ psychological need satisfaction and learning motivation in middle school physical education, although the findings should be interpreted cautiously given the limited sample size, short intervention period, and absence of formal mediation testing.

Overall, the findings provide preliminary evidence that task-based basketball instruction may support middle school students’ psychological need satisfaction and learning motivation. Nevertheless, the results should be interpreted with caution because of the relatively short intervention period, the limited sample size, and the absence of formal mediation testing. Future studies should use larger samples, longer intervention periods, and more rigorous analytical models to further examine the mechanisms through which task-based teaching may influence students’ motivation in physical education.

### 5.2 Limitations

Although this study obtained relatively clear findings, it has the following four Limitations.

First, sample representativeness needs to be improved. This study selected only eighth-grade students from one junior high school in Qingdao. The sample source was relatively limited and did not cover different grades, regions, or teaching contexts in different sports such as football and volleyball. Therefore, the generalizability of the findings requires further verification.

Second, the study variables and measurement period were limited. This study mainly focused on two psychological variables: basic psychological need satisfaction and learning motivation. It did not include outcome variables such as sport skill acquisition, classroom participation, or extracurricular physical activity behavior. In addition, the intervention lasted only 6 weeks, preventing examination of the long-term effects of task-based teaching and sustained changes in motivation.

Third, the depth of mechanism analysis was insufficient. This study preliminarily revealed associations between basic psychological needs and learning motivation through correlation analysis only. However, other statistical tests, including mediation analysis, were not performed to demonstrate the causal relationship between variables and underlying mechanisms therefore are still being studied.

Fourth, the basketball-adapted versions of the instruments were not examined using confirmatory factor analysis. Although the adaptations involved only minor contextual wording changes and the original scales have demonstrated acceptable psychometric properties in previous studies, the present reliability results should not be interpreted as full validation evidence for the adapted versions. Future studies should further examine their factorial validity in basketball learning contexts using larger samples.

### 5.3 Future directions

There are three possible directions for future research: First, research samples could be expanded by involving students of different grade levels, different geographical areas, and different sport projects, in order to determine the external validity of task-based instruction. Second, the duration of the intervention should be lengthened, and 12-week and 24-week follow-up data collection should be included to ascertain whether task-based teaching plays a role in the formation of lasting exercise habits. Lastly, mediation and moderation analyses should be incorporated in future studies to further elucidate the internal mechanisms and external moderators that task-based instruction affects motivation in physical education.

## Data Availability

The raw data supporting the conclusions of this article will be made available by the authors, without undue reservation.
